# Perceived microaggressions in health care: A measurement study

**DOI:** 10.1371/journal.pone.0211620

**Published:** 2019-02-05

**Authors:** Daniel Cruz, Yubelky Rodriguez, Christina Mastropaolo

**Affiliations:** 1 Department of Professional Psychology & Family Therapy (PPFT), Seton Hall University, South Orange, New Jersey, United States of America; 2 Family Medicine Residency Program, Hackensack Meridian Health Mountainside Hospital, Verona, New Jersey, United States of America; University of Arkansas for Medical Sciences, UNITED STATES

## Abstract

The current study examined the psychometric properties of the Microaggressions in Health Care Scale (MHCS), including factor structure, measurement invariance, and internal consistency reliability. We used a cross-sectional research design to study perceived racial microaggressions, discrimination, and mental health in 296 African American and Latino respondents. Participants completed measures that assess healthcare microaggressions and daily discrimination as well as the Depression, Anxiety and Stress Scale (DASS-21). Results revealed that the MHCS has promising psychometric properties. The confirmatory factory analysis (CFA) revealed that the MHCS is a unidimensional scale. Multi-group CFAs provided evidence of measurement invariance across racial / ethnic groups and gender. The internal consistency reliability of the scale was .88 for the overall sample. Microaggressions correlated with daily discrimination scores (*r* = .67), as well as mental health symptoms (*r*’s = .40 –.52). The MHCS is a brief, valid, and reliable measure that can be used to assess and monitor racial and cultural forces that shape patient-provider interactions. This study concludes with a discussion of the ongoing need for research on microaggressions in healthcare as well as implications for future research.

## Introduction

Racial minorities have been pervasively marginalized because of racism and oppression. This social problem has had measurable effects on the health and well-being of people of color and on society as a whole. Racial discrimination is still widespread and is a significant social determinant of minority population health. The health disparities literature suggests that racism is responsible, in part, for the increased morbidity and mortality rates observed among racial minority groups [1]. Furthermore, racial discrimination is understood to be a complicated construct in that it occurs in both obvious as well as subtle ways. For example, researchers have found that racism can occur explicitly through blatant discriminative encounters, as well as through microaggressions [2–3].

Microaggressions are broadly defined as behaviors that ambiguously disempower racial minorities. Microaggressions are brief and subtle verbal and/or non-verbal denigrating messages directed towards ethnic and/or racial minorities that carry the weight of the offending party’s implicit bias, often below their own conscious awareness [4–6]. In response to qualitative analysis and scholarly literature on racism, Sue [3] proposed a taxonomy of racial microaggressions with implications for practice, education and training, and research. The author identified three categories of microaggressions: 1) microassaults, 2) microinsults, and 3) microinvalidations. Microassaults are explicit physical, verbal, or non-verbal acts of racism or discrimination that communicate that the targeted party is of lesser worth. Microinsults are messages that are insensitive and disparaging to a person’s racial identity or background, such as implying that a racial minority did not earn their position. Microinvalidations are behaviors that negate, neutralize, or deny the experiences of People of Color (e.g., racially insensitive comments such as “I don’t see color” and/or “We are all the same”). Despite the sound theoretical framework associated with microaggressions, some researchers argue that empirical testing of this concept remains insufficient [7]. More research is needed however to test the veracity of these arguments given that microaggressions are, by nature, ambiguous, complex, and multidimensional.

The types of prejudice and perceived stigma that are associated with microaggressions have also been found to adversely impact minorities and to be correlated with mental health problems [8–14]. For example, Nadal et al. [15] found that people who had experienced racial microaggressions were more likely than those who had not to report mental health issues such as depression, anxiety, negative affect (or a negative view of the world), and poor behavioral control. In addition, a recent investigation revealed that racial microaggressions were significantly and positively associated with cultural mistrust, which, in turn, related to lower levels of well-being [16].

Healthcare microaggressions refer to implicit discrimination within the healthcare setting, whereby treatment providers who are in positions of authority inadvertently marginalize members of minority groups through culturally insensitive interactions [17]. Healthcare microaggressions originate from the concept of aversive racism; i.e., those in the dominant culture (often in positions of power) deny their prejudices, based on their adherence to egalitarian ideals [2]. More contemporary models propose that healthcare microaggressions represent institutional betrayal, which reflects the systematic organizational practices that fail to respond to discrimination and microaggressions appropriately (e.g., lack of clear surveillance policies or accountability procedures, normalizing culturally insensitive interactions) [18].

Research studies that have examined racial microaggressions in healthcare settings, particularly in the context of medical treatment, are surprisingly limited, with few exceptions. In one study, Smith-Oka used an ethnographic framework to study a group of obstetric patients and uncovered a new form of microaggression, which he termed *corporeal microaggressions* [19]. The term describes the imposed values and potentially hostile treatment and degrading comments made by physicians towards marginalized, pregnant women. More specifically, corporeal microaggressions are defined as “mainstream perceptions of moral superiority and are expressed as violent bodily treatment, such as sterilization efforts that target single mothers” [19]. As an example, the author described physicians who implied that sterilization might be warranted in cases of perceived promiscuous histories among low-income, minority women. The study revealed several other examples of racial microaggressions such as unfair judgments, unfounded assumptions, and biases toward obstetric patients (e.g., interpersonally dominant medical personnel, classicism), all of which placed patients in subjugated and potentially demoralized positions. More research is needed to better understand the concept of corporeal microaggressions beyond qualitative inquiry, including validity testing with Sue’s taxonomy [3] and exploring generalizability.

Microaggressions are subtle, but the consequences are likely additive and, in turn, may create (potentially preventable) barriers to adequate healthcare. Satisfaction with healthcare services has been found to correlate with health, and this association is mediated by the patient-provider relationship [20–22]. Healthcare microaggressions may undermine patient-centered care by threatening the opportunity for a positive relationship to take place. For example, a provider may get frustrated with a patient’s non-compliance with medical treatment recommendations without acknowledging contextual stressors such as lack of adequate health coverage/benefits and potential difficulty navigating complex medical systems and referrals due to limited access to resources. Thus, the patient may feel less supported and fail to follow-up on recommended treatments.

While discrimination has been relatively well studied in the extant literature, research investigating racial microaggressions generally, and their occurrence and potential consequences in healthcare settings specifically, has been limited. Although some studies have shown that racial microaggressions in medical settings are linked to several stress-related health problems (e.g., smoking and pain symptoms [23]), the dearth of research in this area is due, at least in part, to a lack of valid and reliable instruments specifically designed for healthcare settings.

In 2007, Constantine reported on findings from an investigation that examined the impact of racial microaggressions on the mental health treatment of African-American clients [24]. More specifically, clients were interviewed about their experiences working with Caucasian therapists, and their responses were then summarized to capture themes such as minimization of racial-cultural issues in counseling (e.g., undermining the impact of culture in treatment) and overidentification (e.g., clinicians’ assumptions about shared experiences with their clients) [24]. Constantine and her team used the themes to develop the Racial Microaggressions in Counseling Scale (RMCS), a 10-item tool designed to assess perceived microaggressions [24]. The RMCS has demonstrated good reliability, with internal consistency (coefficient alpha) reported at .73. Moreover, results with the tool have revealed that perceived racial microaggressions correlate negatively with treatment satisfaction, therapeutic relationship, and perceived counselor competence [24].

Walls, Gonzalez, Gladney, and Onello [25] adapted the RMCS for a sample of American Indian patients from the Lac Courte Oreilles and Bois Forte Bands of Chippewa tribes who were diagnosed with diabetes mellitus. Walls et al. reported psychometric evidence for a revised, 6-item scale (which they called the Microaggressions in Health Care Scale, or MHCS), including reliability and convergent validity (the measure positively correlated with depression and number of cardiac events). More specifically, the internal consistency coefficient was reported at .85, which was equivalent to that reported for the original RMCS [24]. However, the factorial structure of the MHCS has yet to be evaluated.

The aim of the current study was to provide additional psychometric data for the MHCS with a sample of African-Americans and Latinos in an effort to contribute to the quantitative literature that examines patients’ experience of microaggressions in healthcare. This is a necessary step for the MHCS, given that the measure was adapted from an instrument that was originally intended for use in counseling, and because the revised measure includes only six of the ten original items. On the basis of previous research with both the original and the revised measures, we anticipated that scale items would converge to a single-factor model consistent with a unidimensional structure. We also predicted that patient perceptions of racial microaggressions during visits with healthcare providers would positively correlate with psychological distress. Specifically, higher levels of racial microaggressions would correlate with higher levels of depression, anxiety, and stress. Significant correlations between the MHCS and mental health symptoms would support the measure’s convergent validity.

## Materials and methods

Study recruitment occurred via Amazon’s Mechanical Turk. Potential participants from Mechanical Turk reviewed the study description, and, if interested and eligible, they were directed to a Qualtrics survey. To qualify for the study, participants had to be over the age of 18 and identify as Black/African-American or Hispanic/Latino. After reviewing and agreeing to the informed consent form, participants were directed to the Qualtrics link, where they responded to a demographic questionnaire and completed all psychometric instruments. The study was reviewed and approved by the Institutional Review Board at Seton Hall University.

### Measurement

The Microaggressions in Health Care Scale (MHCS) is a brief, self-report instrument adapted from the Racial Microaggressions in Counseling Scale [24], which was designed to examine participants’ experiences of microaggressions from healthcare providers. The MHCS includes a set of six brief questions on self-reported experiences with microaggressions during interactions with healthcare systems and medical providers. Participants are asked about microaggressions on the following 3-point Likert scale: (1) *this never happened*, (2) *this happened*, *but it didn’t bother me*, and (3) *this happened*, *and I was bothered by it*. Sample items include “avoided discussing or addressing cultural issues” and “sometimes was insensitive about my cultural group when trying to understand or treat my issues.” The measure has shown preliminary psychometric support, primarily through a reported reliability coefficient of .85 and significant correlations with health outcomes [25]. Copies of the RMCS and the MHCS are available from the study authors [24–25].

The Depression, Anxiety and Stress Scale [26] is a 21-item, self-report measure designed to assess depression, anxiety, and stress. Participants are asked a series of questions and respond using the following 4-point Likert scale: (0) *Did not apply to me at all*, (1) *Applied to me to some degree or some of the time*, (2) *Applied to me to a considerable degree*, *or a good part of time*, and (3) *Applied to me very much*, *or most of the time*. The validity and reliability of the measure was presented by the authors, who administered the DASS-21 to a large sample of 717 participants. A confirmatory factor analysis (CFA) supported a three-factor solution which accounted for a significant percent of the item variance (i.e., exploratory factor analysis resulted in 41.3% of explained item variance). Participants in that study also completed similar, well-established measures for convergent validity evidence including the Beck Anxiety Inventory (BAI) and the Beck Depression Inventory (BDI). The DASS-Anxiety and DASS-Depression subscales strongly correlated with the BAI (*r* = .81) and the BDI (*r* = .74), respectively. Moreover, the authors reported satisfactory coefficient alphas for Depression (α = .91), Anxiety (α = .81), and Stress (α = .89) [26].

The Everyday Discrimination Scale Adapted to Medical Settings [27–28] measures discrimination in healthcare environments. There are a total of seven questions on this measure, including “a doctor or nurse acts as if he or she thinks you are not smart,” “a doctor or nurse acts as if he or she is better than you,” “you are treated with less courtesy than other people,” and “you feel like a doctor or nurse is not listening to what you are saying.” Participants respond on a 5-point Likert scale from *Never* to *Always*. The Cronbach’s alpha for this instrument was reported to be .89, indicating strong internal consistency [27].

### Data analytic plan

Data analysis consisted of examining the measure’s descriptive statistics, testing parametric assumptions, and evaluating the internal consistency and factor structure using CFA. For the CFA, following the recommendations of Kline [29], several goodness-of-fit indices were evaluated to determine model fit. More specifically, the model chi-square, Root Mean Square Error of Approximation (RMSEA), Comparative Fit Index (CFI), the Tucker-Lewis Index (TLI), and the Standardized Root Mean Square Residual (SRMR) were evaluated. According to Hu and Bentler, the following values indicate a good fit: RMSEA close to .06 or below, CFI close to .95 or greater, TLI close to .95 or greater, and SRMR close to .08 or below [30].

Measurement invariance of the MHCS was evaluated across gender and race/ethnicity by testing models in three steps. In the first step, configural invariance was examined by specifying the same unidimensional factor structure for all groups, but allowing loadings, intercepts, and residuals to differ across groups. After establishing equivalent factor structure across groups, in the second step, metric invariance was assessed by constraining factor loadings to equality. In the last step, and after establishing equivalence of factor loadings, scalar invariance was evaluated by constraining intercepts to be equal across groups. For each test of invariance, change in chi-square, Comparative Fit Index, and Root Mean Square Error of Approximation were used to determine if the constraints at each step reduced model fit. Significant changes in chi-square, CFI > .02 and RMSEA of .03 indicate reduced model fit. Both CFA and invariance tests were performed in R version 3.5.1 [31] using the package Lavaan [32]. Full information maximum likelihood (FIML) estimation was used to handle missing data. Once measurement validity and reliability were established, Pearson correlations were computed for the main study variables, followed by MANOVAs to test participant microaggression scores for mean (Bonferroni corrected) group differences on demographic variables.

## Results

Participant demographic variable analysis revealed that the gender composition of the participants was relatively equal (51% male and 49% female; Table 1). The average age of participants was 35 ± 10.91 years (range: 18 to 71). The majority of participants were college educated (89%), worked full-time (72%), and earned between $21,000 to $60,000 per year (56%).

**Table 1:** Participant characteristics.

Demographic Variables	*n*	*%*
Race/Ethnicity		
Black/African American	188	64
Hispanic/Latino	108	36
Sex		
Male	150	51
Female	146	49
Education		
High School Graduate	32	11
Partial College	129	44
College Graduate / Post-Baccalaureate	135	45
Employment		
Full-time	212	72
Part-time	44	15
Unemployed	37	13
Annual Income		
≤ $20,000	63	21
$21,000-$40,000	100	34
$41,000-$60,000	66	22
$61,000-$80,000	37	13
$81,000+	30	10

The mean microaggression total score was slightly higher in this study (*M* = 1.6, *SD* = .60) than in the original study by Walls et al. [25], though the difference is not statistically significant (z = +0.26, *ns*). Means, standard deviations, and standardized factor loadings are shown in Table 2 for each scale item. The item means ranged from 1.48 to 1.63 and standard deviation values from 0.67 to 0.86. Skewness and kurtosis values were all within normal limits in that no value exceeded a 1.96 critical cutoff [33]. Internal consistency for the MHCS was strong (α = .88) and in line with previous findings [25]. The one-factor CFA model of MHCS fit the data well (Chi-square (9) = 13.832, *p* = .128 CFI = .992, TLI = .987, RMSEA = .051, and SRMR = .021). This confirms that the MHCS has unidimensional factor structure with all six items loading on one latent factor.

**Table 2:** Descriptive statistics and factor loadings.

My healthcare provider:	*M*	*SD*	Standardized Factor Loadings
Avoided discussing or addressing cultural issues	1.56	0.67	.506
Sometimes was insensitive about my cultural group when trying to understand or treat my issues	1.61	0.86	.649
Seemed to deny having any cultural biases or stereotypes	1.51	0.84	.638
At times seemed to over-identify with my experiences related to my race or culture	1.50	0.82	.542
At times seem to have stereotypes about my about my cultural group, even if he or she did not express them directly	1.63	0.85	.639
Sometimes minimized the importance of cultural issues	1.48	0.82	.600

Significant factor loadings were defined as loadings ≥ .40

Next, three types of measurement invariance for the MHCS was evaluated for gender and race/ethnicity: configural, metric, and scalar. With regard to gender, configural invariance was reasonably supported (Table 3). This means that the organization of the factor structure (i.e., six items loading on a latent factor) is supported. Although chi-square change values were statistically significant for both metric and scalar invariance models, change in CFI and RMSEA fell well below the threshold outlined by Rutkowski and Svetina [34] in support of metric and scalar invariance models. More specifically, the authors recommend cutoff values of .030 and—.020 for change in CFI and RMSEA, respectively.

**Table 3:** Test of measurement invariance by gender.

Invariance Model	Chi-square (*df*)	CFI	RMSEA	Chi-square Change (*df*)	CFI Change	RMSEA Change
Configural	35.382(18)	.970	.106	NA	NA	NA
Metric	42.604(23)	.966	.100	7.22(5)*	.004	.006
Scalar	54.676(28)	.954	.105	12.072(5)*	.012	.006

* *p* < .05

Measurement invariance test results by race/ethnicity show that the configural model fits the data well, suggesting the factor structure of the MHCS is equivalent across the two racial/ethnic groups (Table 4). The chi-square change for metric and scalar invariance models were both non-significant. In addition, the decrement in CFI and RMSEA change are well below the thresholds indicated above (i.e., .030 and -.020). This suggests that the contribution of each items to the latent factor are similar across both racial/ethnic groups and that latent mean differences are reflective of shared variances in observed items.

**Table 4:** Test of measurement invariance by race/ethnicity.

Invariance Model	Chi-square (*df*)	CFI	RMSEA	Chi-square Change (*df*)	CFI Change	RMSEA Change
Configural	26.881(18)	.984	.076	NA	NA	NA
Metric	35.898(23)	.977	.081	9.016(5)*	.007	.005
Scalar	41.662(28)	.975	.075	5.764(5)*	.001	.005

* *p* < .05

### Correlations

Pearson correlations (1-tailed) were computed to test for relationships among the variables of interest. More specifically, the relationship between discrimination and racial microaggressions was examined using the computed index (total) score (range = 0–12). Racial microaggressions positively correlated with discrimination (*r* = .67, *p* < .01). Participants that reported higher levels of perceived racial microaggressions also reported more psychological distress symptoms. This was observed through positive correlations between racial microaggressions and DASS-21 scores for stress (*r* = .45, *p* < .01), anxiety (*r* = .52, *p* < .01) and depression (*r* = .40, *p* < .01).

### Demographic analysis (MANOVAs)

Racial microaggression scores did not differ between Black/African-American (*M* = 2.4, *SD* = 2.3) and Hispanic/Latino participants (*M* = 2.2, *SD* = 2.3), *t* (203) = .33, *p* = .75. In addition, no significant differences were found when comparing racial microaggressions between men (*M* = 2.3, *SD* = 2.3) and women (*M* = 2.4, *SD* = 2.3), *t* (203) = -.34, *p* = .73. Participants with some college experience reported significantly higher levels of perceived microaggressions (*M* = 2.7, *SD* = 2.3) compared to those that either had a high school level of education (*M* = 2.6, *SD* = 2.60), or completed college and/or obtained post baccalaureate training (*M* = 1.8, *SD* = 2.2), *F*(2, 202) = 3.66, *p* = .03, Eta squared = .035.

The ANOVA for employment status (full-time, part-time, unemployed) and microaggressions was significant, *F*(2, 202) = 4.61, *p* = .01, Eta squared = .044. Post hoc mean comparisons with Bonferroni correction revealed differences between participants that were employed full-time versus part-time and part-time versus unemployed. Specifically, participants that were employed on a part-time basis reported significantly more perceived microaggressions (*M* = 3.31, *SD* = 2.2) than those that were employed full-time (*M* = 2.16, *SD* = 2.27) and those that were unemployed (*M* = 1.76, *SD* = 2.28). Income was not significantly associated with microaggressions, *F*(4, 202) = 1.20, *p* = .31.

## Discussion

The primary goal of this study was to provide psychometric evidence for the Microaggressions in Health Care Scale with a sample of African-Americans and Latinos. The confirmatory factor analysis showed strong evidence in support of a single-factor solution for the instrument. Moreover, measurement invariance testing revealed good model fit across all multigroup comparisons and within increasingly constrained models for both gender and race/ethnicity.

This study’s findings are consistent with those previously reported by Walls and colleagues [25] and suggest that microaggressions may pose significant risk to the quality of life for People of Color. Microaggressions positively correlated with reports of perceived discrimination. This provides evidence of the measure’s convergent validity and suggests that People of Color are at risk of facing multiple forms of discrimination by healthcare providers, which likely carry additive health consequences. It has been suggested that racial microaggressions are especially detrimental when enacted by health care providers [25] and that the negative impact of racial microaggressions is especially salient in medical settings where physicians hold significant authority over the individuals they treat. Furthermore, patients tend to be in vulnerable states when seeking medical treatment. As a result, they may be especially susceptible to psychological distress in response to microaggressions, which likely compounds their medical problems. Unlike in counseling settings, patients will likely not have the opportunity to process these feelings with their physicians because of the limited time and the general training culture of medicine, which tends to emphasize standardized medical procedures and efficiencies.

Many participants in this study felt that their doctors held negative stereotypes about their racial/cultural groups. Participants reported that physicians were culturally insensitive and/or that they avoided addressing diversity in their medical encounters. These findings suggest that People of Color are highly attuned to the racial/cultural contexts that take place in their interactions with healthcare providers, likely because of past experiences with racism as a chronic stressor that is not limited to isolated interactions. More specifically, racism is embedded in the collective unconscious of society and is transmitted through systemic and institutional practices. As such, denial of cultural differences by physicians or avoidance of discussions related to individual differences only reinforces the oppressive ideology that is at the core of maintaining the status quo, which continues to place racial minorities in a subjugated position.

African-Americans and Latinos suffer consistent health disparities, and the stress associated with discrimination is now understood to be a critical component of these issues. Microaggressions are unconscious and detrimental barriers to health and well-being. The current study found that racial microaggressions correlated with depression and anxiety as well as with psychological stress. These findings are consistent with previous research that found evidence to suggest that negative health symptoms, such as depression and anxiety, manifested from the strain associated with experiencing racial microaggressions [12, 25].

This study found significant differences in microaggressions by education and employment history. More specifically, participants that reported some college exposure reported higher levels of perceived healthcare microaggressions than participants that either completed high school or college. Analyses also revealed that respondents that were employed part-time at the time of the study reported higher microaggression scores compared to individuals that worked full-time or were unemployed. It is possible that participants with some college exposure and/or who worked part-time may have experienced institutional discrimination or received disparaging messages in these contexts which, in turn, served as a barrier to educational or occupational achievement; therefore, these participants were less likely to benefit from the status afforded to those with higher levels of achievement.

Microaggressions operate implicitly in the patient-physician relationship and will likely stay unrecognized unless an ongoing intentional, reflective, and process-oriented practice is implemented. Examining microaggressions should be viewed as a growth promoting, educational opportunity that has the potential to improve individual interactions and system level practices. To work toward patient-centered and culturally informed practice, healthcare providers should complement didactic training to include experiential training on the fluid and intersecting group memberships with which their patients may identify. It may be counterproductive to define multicultural competency with dichotomous racial categories, therein perpetuating and reinforcing stereotypes [35], which may then impede providers’ ability to recognize the moment-to-moment thoughts, feelings, and behaviors that may be inadvertently disempowering their patients. Kleinman and Benson [36] state that to raise the consciousness of providers when working with diverse individuals, they must “[…] empathize with the lived experience of the patient’s illness, and try to understand the illness as the patient understands, feels, perceives, and responds to it.” This reflective practice must also include a holistic appreciation for the individual, an understanding of the oppressive nature of institutions against People of Color, and the acknowledgment that the provider is in a position of power and privilege. The aforementioned suggestions hold promise in that they have the potential to significantly improve patients’ quality of life and reduce the health disparities that unjustly affect African-Americans and Latinos.

The current study has several limitations worth noting. First, the study asked participants to identify as either Black/African-American or Hispanic/Latino. This categorical approach assumes that an individual belongs to one of the two groups, when, in fact, some may identify as multiracial. Future studies should examine to what degree dimensions of racial identity may better explain the variance and impact of perceived microaggressions in healthcare settings and on health outcomes. In addition, data for the current study were obtained from an online recruitment program, and the sample included mostly college educated individuals who worked full-time. This may limit the generalizability of this study’s findings. Future research studies should obtain larger and more diverse samples and expand the sample to include other racial and ethnic groups. Moreover, future studies should underscore racial and ethnic identity as strength-based protective factors, including spiritual and religious resources and family and community connectedness.

## Conclusions

Accurate assessment and identification of racial microaggressions, particularly within the healthcare environment, is critical for improving health outcomes in vulnerable populations.

The current study presented additional evidence to support the use of the Revised Microaggressions in Health Care Scale for this purpose. Continued work to develop and validate microaggression measurement scales for use within healthcare environments holds potential to provide more empirically sound findings. This, in turn, will allow us to more accurately and comprehensively identify and understand patients’ experiences and to work towards providing better healthcare to patients of all backgrounds.
